# The impact of patient engagement on trials and trialists in Ontario, Canada: An interview study with IMPACT awardees

**DOI:** 10.1186/s40900-022-00381-7

**Published:** 2022-09-07

**Authors:** Stuart G. Nicholls, Grace Fox, Zarah Monfaredi, Evelyn Poole, Chantelle Garritty, Alies Maybee, Justin Presseau, Beverley Shea, Dean A. Fergusson

**Affiliations:** 1 Clinical Epidemiology Program, Ottawa Hospital Research Institute, Ottawa, ON Canada; 2 School of Epidemiology and Public Health, University of Ottawa, Ottawa, Canada; 3 Faculty of Health Sciences, Simon Fraser University, Burnaby, Canada; 4 Faculty of Arts and Science, Queen’s University, Kingston, Canada; 5 Global Health and Guidelines Division, Public Health Agency of Canada, Ottawa, Canada; 6 Patient Partner, Toronto, Canada; 7 Faculty of Medicine, University of Ottawa, Ottawa, Canada

**Keywords:** Patient engagement, Qualitative research, Evaluation, Representation, Impact

## Abstract

**Background:**

A key component of patient-oriented research is the engagement of patients as partners in the design and conduct of health research. While there is now national infrastructure and networks to support the engagement of patients as partners, there remain calls for promising practices and success stories. In particular, there remains a keen interest in evaluating the impact that patient engagement has on health research studies. We aimed to investigate the impact that patient engagement had on health research conducted in Ontario, Canada.

**Methods:**

Our sampling frame was studies that were awarded funding by the Ontario SPOR SUPPORT Unit. Semi-structured interviews were conducted with 10 principal investigators, members of research teams, and patient partners. Interviews explored the role of patient partners, the perceived impact of the patient engagement on the study, challenges faced, and advice for other researchers considering patient engagement. Data were analysed using the thematic analysis method with transcripts coded independently by two members of the study team. All coding and subsequent theme generation were discussed until consensus was achieved.

**Results:**

There was variation in the methods used to engage patients and other stakeholders, the roles that patients and stakeholders occupied, and where they had input. Interviewees discussed two major areas of impact of patient engagement on research: impact on the study about which they were being interviewed, which tended to relate to improved relevancy of the research to the study population, and impact on themselves which led to changes in their own practice or approaches to future research. Identified challenges to patient engagement included: identifying and reaching patient advisors or patient partners, time-related challenges, and maintaining engagement over the course of the research.

**Conclusions:**

There remains a need to further build out the concept of relevancy and how it may be operationalised in practice. Further, the longer-term impacts of patient engagement on researchers and research teams remains under-explored and may reveal additional elements for evaluation. Challenges to patient engagement remain, including identifying and maintaining engagement with partners that reflect the diversity of the population of interest.

**Supplementary Information:**

The online version contains supplementary material available at 10.1186/s40900-022-00381-7.

## Plain English summary

We interviewed 10 individuals associated with studies that were funded through the Ontario Strategy for Patient Oriented Research (SPOR) Unit. We asked them about how they had engaged patients, families or caregivers in their study, how this engagement had influenced their study, the challenges they faced, and advice for other researchers considering engaging with patients, families and caregivers. We found that patients had been engaged in a variety of ways and at different times. The changes brought about by the patient engagement varied between studies but tended to reflect an improvement in how relevant the study was to patients. Researchers also shared how engagement with patients, families, and caregivers had changed their approaches to future research. Key challenges facing researchers included finding partners that were appropriate for the study, as well making sure that the approach to engagement overcame issues such as financial or logistical barriers that could prevent patients, families or caregivers from partnering with the study.

**Supplementary Information:**

The online version contains supplementary material available at 10.1186/s40900-022-00381-7.

## Introduction

Patient-oriented research—a continuum of research designs that focus on questions and outcomes relevant to patients—[1] has become an important part of health research [2]. The conduct of patient-oriented research is increasingly supported by funding, methodological support, and training programs at global, national and institutional levels. Established organisations include INVOLVE in the UK and the Patient-Centered Outcomes Research Institute (PCORI) in the US. In Canada, the Canadian Institutes of Health Research (CIHR) established the Strategy for Patient-Oriented Research (SPOR) in 2013. There have been numerous calls for funding through SPOR, a nationally mandated curriculum for patient-oriented research, as well as training for researchers, trainees, and patient partners in the foundations of patient-oriented research. In addition, SPOR funding supported the creation of centres of excellence that champion the development of capacity in patient-oriented research as well as providing resources and training to research teams.

A key component of patient-oriented research is the engagement of patients as partners in the design and conduct of health research, or what is more commonly referred to as “patient engagement”. In the Canadian context of SPOR the term “patient” is used in a very broad sense and is defined as being “inclusive of individuals with personal experience of a health issue and informal caregivers, including family and friends” [3].

While there have been concerted efforts to create dedicated infrastructure and networks to support patient-oriented research, and numerous examples of funded work, there remain calls for promising practices and guidance in the form of ‘success stories’ and illustrations of patient engagement at work [4–6]. In particular, there remains a keen interest in evaluating the impact that patient engagement has on health research studies [7].

In 2013, INVOLVE published a report entitled, *Exploring the impact of public involvement on the quality of research: Examples* [8]. This report brought together researchers’ perspectives on public involvement^1^ in research from six INVOLVE-affiliated studies aiming to ascertain the ways in which public involvement influenced the quality of their research. In the present study our aim was to take a similar approach in order to develop knowledge regarding the impact that patient engagement has had on research conducted within the Canadian context.

## Methods

To understand the impact that patient engagement had on research, we undertook semi-structured telephone interviews with members of teams awarded funding to directly support patient-oriented research and patient engagement. The study is reported in accordance with the Standards for Reporting Qualitative Research (SRQR) reporting guideline [9].

Our sampling frame was studies supported by the Ontario SPOR SUPPORT Unit (OSSU) Innovative, Measurable, Patient-oriented, Appropriate, Collaborative, and Transformative (IMPACT) award. OSSU [10], established in 2015 through CIHR SPOR and Ontario government funding, is a dedicated network of 15 leading health research centres in Ontario that engages researchers, patients, decision-makers, and other partners in patient-oriented research to improve the health of Ontarians and the health care system. OSSU provides supports and resources to people conducting patient-oriented research to help implement Canada’s SPOR in Ontario. The IMPACT Awards were announced in November 2014. All applications had to involve at least three OSSU research centres, which were to provide expertise and resources to teams conducting patient-oriented research, and had to engage patients as study partners or advisors. Funding for successful proposals began in June 2015 and prior to conduct of this study, there had been no attempt to report on the patient engagement component in these studies.

Individuals were eligible for interview if they had served as a principal or co-investigator, a member of the research team (for example, a research coordinator), or as a patient partner or advisor from teams awarded an OSSU IMPACT award.

### Identification and recruitment

A total of 17 projects were funded in various areas of health research. To identify interviewees, we identified the IMPACT Award-winning principal investigators (PIs) and their projects from publicly available information. Specifically, we identified projects that were published on the OSSU website (www.ossu.ca) and which were subsequently featured in a special supplement of the Canadian Medical Association Journal (https://www.cmaj.ca/content/190/supplement). Contact details for each PI were obtained from the published contact information within the CMAJ supplement or their institutional webpages where this was not possible.

Each PI was contacted by email, invited to participate in the study, and asked to identify a research co-ordinator or member of the research staff and a patient partner from the IMPACT awarded study for our team to contact. Research co-ordinators and patient partners were contacted by email if it was provided by the PI.

### Data collection

Data was collected through a case study-based approach using interviews with the PI, members of the research team, and patient partners where possible. Each interview followed a semi-structured topic guide, allowing for flexibility while covering key topics. The interview guide was influenced by prior work undertaken in the UK by INVOLVE [8], but broadly covered a description of the study, whether the patient engagement approach was informed by a theory or framework, the role of patient partners or advisors, the perceived impact of the patient engagement on the study, challenges faced, and advice for other researchers considering patient engagement. The full interview guide is included as Additional file 1: Interview guide. All interviews were conducted by one member of the team (ZM) in her role as patient-oriented research program facilitator at the Ottawa Hospital Research Institute, with a subset assisted by a second member of the study team (EP).

Given the dispersion of award winners across the province, interviews were conducted by telephone and audio recorded with consent. All audio recordings were transcribed verbatim by a transcription service and at which point they were de-identified. Each transcript was assigned a unique reference number. All transcripts were uploaded to qualitative data analysis software (NVivo 11, QSR International) [11] prior to the start of analysis.

### Data analysis

Transcripts were analysed using the thematic analysis method outlined by Braun and Clarke [12]. Thematic analysis is a manner of identifying, analysing, and reporting patterns, or themes, within the data set [12]. Importantly, a systematic and disciplined thematic analysis enables a more accurate and sensitive understanding of a given topic [13]. This is fitting for the present study due to the subjectivity of engaging patients in research and describing that experience. The thematic analysis was performed inductively, meaning codes were developed from the data and not by fitting the data into preconceived ideas or themes. Further, the analysis identified semantic themes through interpretation of the discourse directly [12]. To enhance trustworthiness and credibility of the analyses, a codebook was maintained throughout the study. All transcripts were coded independently by two members of the study team (SN and GF) who met after analysing each transcript and discussed coding until consensus was achieved. During this process annotations were made to record evolving ideas or potential thematic groupings of individual codes. Following the completion of the initial open coding, the two reviewers developed thematic groupings through discussion. These themes were presented to the larger team, including the patient co-author, and revised based on feedback. This iterative process continued until a general consensus developed.

This study protocol was approved by the Ottawa Health Science Network Research Ethics Board (Ref: 20170691-01H). All participants provided individual consent to participate.

## Results

Of the 17 funded studies, participants from 7 studies agreed to take part with a total of 10 interviews. Interviews took place in July and August 2019, lasting on average 49 min in duration (range 29–66 min). Of the 10 interviewees, one was a patient partner, three were research staff, and six were investigators. All studies involved conducting a clinical trial.

Interviewees varied with respect to their experience with patient engagement; for some, this was their first project engaging patients or other stakeholders, while others had developed long-standing relationships with patients and/or stakeholder groups.

There was variation in the methods used to engage patients and other stakeholders, and the roles that patients and stakeholders occupied and where they had input. The number of patients engaged within studies also varied. For example, one study reported a single patient partner, others reported four patient partners, while another reported eight participants engaged in focus groups and between 10 and 14 parents that participated in the advisory panel. Others still indicated they had received input from hundreds of stakeholders.

Theory informed approaches were rare with respect to the ways in which the theory informed patient engagement within the study. For example, one participant referred to a framework set out by the Ontario SPOR Support Unit (OSSU) that informed their engagement approach, while others indicated that their work was not grounded in any particular theory. Other interviewees discussed frameworks that informed their overall research program, but were not specific to patient engagement, for example, the Access Framework developed by Levesque et al. [14], the McCain Model of Youth Engagement [15], or the Knowledge-to-Action process [16].

We present our thematic findings below. Specifically, we present these within descriptive accounts of three key areas of focus within the interviews: (1) approaches to patient engagement; (2) areas of impact, noting the impact that patient engagement had on the specific study in question but also on future research, and; (3) the identified challenges and noted approaches to ameliorate these. Illustrative quotes are provided within the text, with additional quotes provided in Table 1.

**Table 1:** Additional quotes

Topic	Quote
*3.1 Methods and roles*	3.1.1 “There were eight participants in our focus groups that we've engaged specifically around the pilot—the parent pilot study. The first focus groups have been eight participants, and we also alongside have what we call the parent panel, where we have anywhere between 10 and 14 parents that participate in the advisory panel. These are the two ways we have engaged families directly around how this works.” *P1, Principal Investigator* 3.1.2 “we've committed to hiring people who have lived experience onto our team, so that on a daily basis they're involved in all decisions, and they weigh in on every decision.” *P7, Principal Investigator* 3.1.3 “[…] as far as the advising part of the development—so that's where we were asking about the different questionnaire—the different questions and the different items. That was done via email, sending it to participants that expressed interest in developing it, and that was simply sent to individuals who would then—and then it was and Excel sheet where they could write in all their comments and then send that back to us.” *P2, Research Staff* 3.1.4 “I think one of the things we found out early on was that these families were very engaged via Facebook, and so we decided to try to meet them there […] We created a Facebook page. […] We're seeing some success there as far as making Facebook the go-to area for connecting with these families. I think that in and of itself, people have been very responsive, and it's really great to be able to interact with the families in, I think, a mode that they're on anyways. We're not asking them to get out of their usual-ish schedule, which is really nice.” *P2, Research Staff*
*3.2 Impact of patient engagement*	
3.2a Impact on the study	3.2a.1 “A primary outcome measure is functioning, which as a youth contribution, one—the psychiatrist sitting out around a table, and healthcare providers were automatically defaulting to symptoms. Then, they went and talked to youth, and they're, no, it's not symptoms I'm interested in. I want to know how I'm doing on a daily basis. How's it going at school? How's it going with my friends? How's it going among my family? Primary outcome measures, that's a pretty serious decision. That was youth led.” *P8, Research Staff* 3.2a.2 “Our quality of life measures have come from some medical world of you had a heart attack, and now we're—can you go up and down the stairs? These are things that are—that often are completely irrelevant to young people who are, ‘I feel like killing myself because my boyfriend broke up with me, and my parents are telling me it's no big deal, and now I'm going to fail my math course, because I didn't study for the exam’.” *P7, Principal Investigator* 3.2a.3 “Yes [it has made a difference to the outcomes], because it's actually what youth wanted. It's actually relevant to our experience, because yeah maybe you might not necessarily, you can still be depressed, but if you're not going to school or something, is that helpful, or—do you know what I mean?” *P6, Patient partner* 3.2a.4 “Some of the feedback we've received around time of day, location, their interest in using technology, as opposed to coming in person. Some of those implementation questions have been—it's been very useful having parents provide us feedback there.” *P1 Principal Investigator* 3.2a.5 “Patients were involved on the [group] in terms of developing—okay, for example, what does a navigator do? What are their qualifications? If we're actually going to get the referral form to the patient, the patients told us, these are things that we'd like to see on it. These are the issues you need to be aware of with respect to health literacy and so on. They were involved in the consultation phase, in the development of the intervention, at every [group] meeting during the implementation, and so it began to tweak things as it rolled out.” *P4, Research Staff* 3.2a.6 “I think in terms of this case, they have been involved right from the start of the production of the knowledge translation tools which were used in this study, and then parents are investigators on the study, and have been at the meetings, and have been interviewed with me.” *P5, Principal Investigator*
3.2b Impact on the researcher	3.2b.1 “I think part of that success was really just being open to the idea of doing research differently, and for good reason, to push ourselves as researchers who have been involved in lots of projects over the years, but perhaps not with this level of emphasis and intentional design around engaging patients. I think having been through it, I can never go back. I think it just makes the research so meaningful.” *P9*, *Principal Investigator* 3.2b.2 “I certainly think that this population and interacting with them has opened my eyes to so many things, both in research and just in life […] it really can change your lens on how you view research, how you view your life, and may certainly – those participants have really opened my eyes to the reality of what it's like to have a child with medical complexity. Of course, I'll never have the understanding of it, because I've not lived it. But I certainly have a very deep appreciation for those parents, and that was through the patient engagement. I think it's one thing to read it on paper, but when you're interacting with people, it just—there's a difference—a significance, and it really makes you understand so much more what we're dealing with.” *P2, Research Staff* 3.2b.3 “This is something that we've developed far more for other initiatives since them, so essentially you can say that SPOR helped us understand the value and benefit of engaging with patients, and so with the other projects we've done things much better.” *P3, Principal Investigator* 3.2b.4 “And that's one thing actually, we did tell people like I always say to them, like, we're learning. I mean, if do things that are wrong, you tell me. Don't assume that, you know, there would that there's, like, I don't necessarily know how to do this. Well, so we're both learning. You're learning research and learning this partnership.” *P10, Principal Investigator*
*3.3 Challenges*	
3.3a Identifying and reaching patient advisors and patient partners	3.3a.1 “For example, if the study was on a chronic disease, and there were community organizations around that chronic disease, then I could see that our involvement in engagement of their patients, and I could see how that community organization would be really directly key to the patient engagement. If we're talking about healthy children, a little bit more of that, community organisations that represent healthy children is a little bit, struggling to think about those.” *P1, Principal Investigator* 3.3a.2 “Then, it's like ensuring that you're not just looking for young people to say what you want to hear, but you're actually—you should be almost more interested in the things if they don't fit with what you already thought. That represents a different point of view.” *P7, Principal Investigator* 3.3a.3 “Yeah, and so again, there's more work coming out about how we should pay patient partners, because when we get funding, we don't pay out Co-I’s. It's our job, but then again, it is our job, whereas their family. Certainly, we always pay gas, and childcare, and I don't—coffee carts, meal carts. To pay them money? I've never put that in a grant yet, and what should that be? What should an honorarium for a patient partner be? […] Yeah, so we're still working that out, and that's—I don't know. I don't have the answer.” *P5, Principal Investigator* 3.3a.4 “I don't know if this has come up in your process to date, but even—I know there's a lot of discussion out in the patient engagement world just around sort of how do we properly recognize these essential partners in research? Is it—what are we doing to do that? Does compensation need to come into it, and how do we make sure that people are being compensated and recognized in a way that is most appropriate for their oftentimes very involved contribution?” *P9*, *Principal Investigator*
3.3b Time-related challenge	3.3b.1 “You've got to move a little bit slower sometimes.[…] We knew that we were going to be doing this from the get go, so it was worked into our timeline. I don't think that was a as big a deal as much as getting REB approval, some of the other stuff. This is a slow process anyways. It wasn't such a major barrier. Sometimes you just have to relax a little bit and take your time on making decisions, but do it anyway, because of institutional mistakes.” *P8, Research Staff* 3.3b.2 “I think for us particularly with the population that we are trying to engage with, like I mentioned, they just don't have a lot of time, which is absolutely fair, since they are—they're very busy managing their child's health, managing their family, managing their own life. It's—the challenge is trying to engage them, even though they don’t have a lot of time, […].” *P1, Principal Investigator* 3.3b.3 “I think it's true for everyone, but my area's youth based, see as being especially important to youth, because the demands on their time, their developmental context, the tasks with which they are confronted over time, and so they may really want to be involved, but if they're in the middle of their grade 12 year where they're really focused on trying to get good grades to get into university, it's not appropriate to ask them to make a big commitment.” *P7, Principal Investigator* 3.3b.4 “[Interviewer: Yeah. Yeah, and how do you build that rapport?] Being open, a little bit, giving a little bit of time out to chat with them about what matters to them. Inviting conversation, not being the ivory tower dry scientist that they can come down to a real person's level.” *P8, Research Staff* 3.3b.5 “Sometimes it was a matter of meeting with them individually and talking about what's coming up at the meeting, but also providing really practical supports, like ensuring transportation, taxi, if they needed it. Made sure that all of our meeting rooms were physically assessable…” *P4, Research Staff* 3.3b.6 “I can't say that this [relationship] is something that was built for that project. This is something that we built over time that previously existed and we just continued to nourish so to speak.” *P3, Principal Investigator*
3.3.c Maintaining engagement	3.3c.1 “We really from the very beginning were sitting together, and the youth changed over time, […] our youth became not youth. At some point, you're, oh right, you are now 10 years older…and the youth were trying to understand that you're actually no longer youth.” *P7, Principal Investigator* 3.3.c.2 “I think some of the challenges we've had, it's a long project and with the randomized control trial nature of it, there's a lot of protection and oversite with the data to ensure the credibility of the trial. I think for me, maybe that it's something that we would want to consider moving forward is just sort of the lulls in time in that data collection phase where there's passion and energy from patients to be engaged, and then it's just trying to think collectively between all of us on the project, including the family partners and family leaders. What are those components that this kind of data collection where we can all continue to be excited and engaged along the way.” *P9, Principal Investigator*

### Approaches to patient engagement

The populations engaged as partners within the individual studies varied, with studies engaging children and youth, young adults, parents of young children, adult patients, community members, and healthcare professionals and community organisations. The breadth of stakeholder groups engaged was reflected in the myriad ways in which interviewees discussed the roles of the patients and stakeholders. Interviewees discussed varied approaches to engaging their patient partners, with these approaches tailored to the populations engaged. Some reflected on how their study had been co-created, and thus involvement had been throughout. In other instances, roles or engagement opportunities focused upon certain aspects of the study, such as helping identify the appropriate outcome, or reviewing study material.

Examples of these patient engagement methods included: the creation of advisory panels or committees that fed back on the study design or intervention; focus groups to elicit ideas or feedback (Quote 3.1.1); and even hiring individuals who were patients or who represented the target population for the study and embedding them within the research team (Quote 3.1.2). Engagement mechanisms included in-person meetings, email (Quote 3.1.3), or sometimes via social media (e.g. utilising existing Facebook groups to engage networks of patients) (Quote 3.1.4).

### Impact of patient engagement on the research

**Fig. 1:**
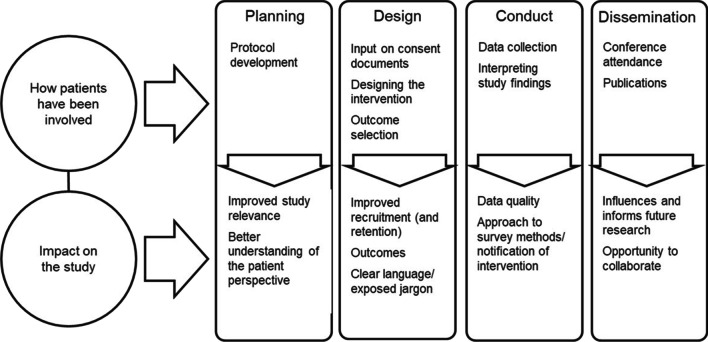
Examples of how patients had been involved and impact on the study

**Fig. 2:**
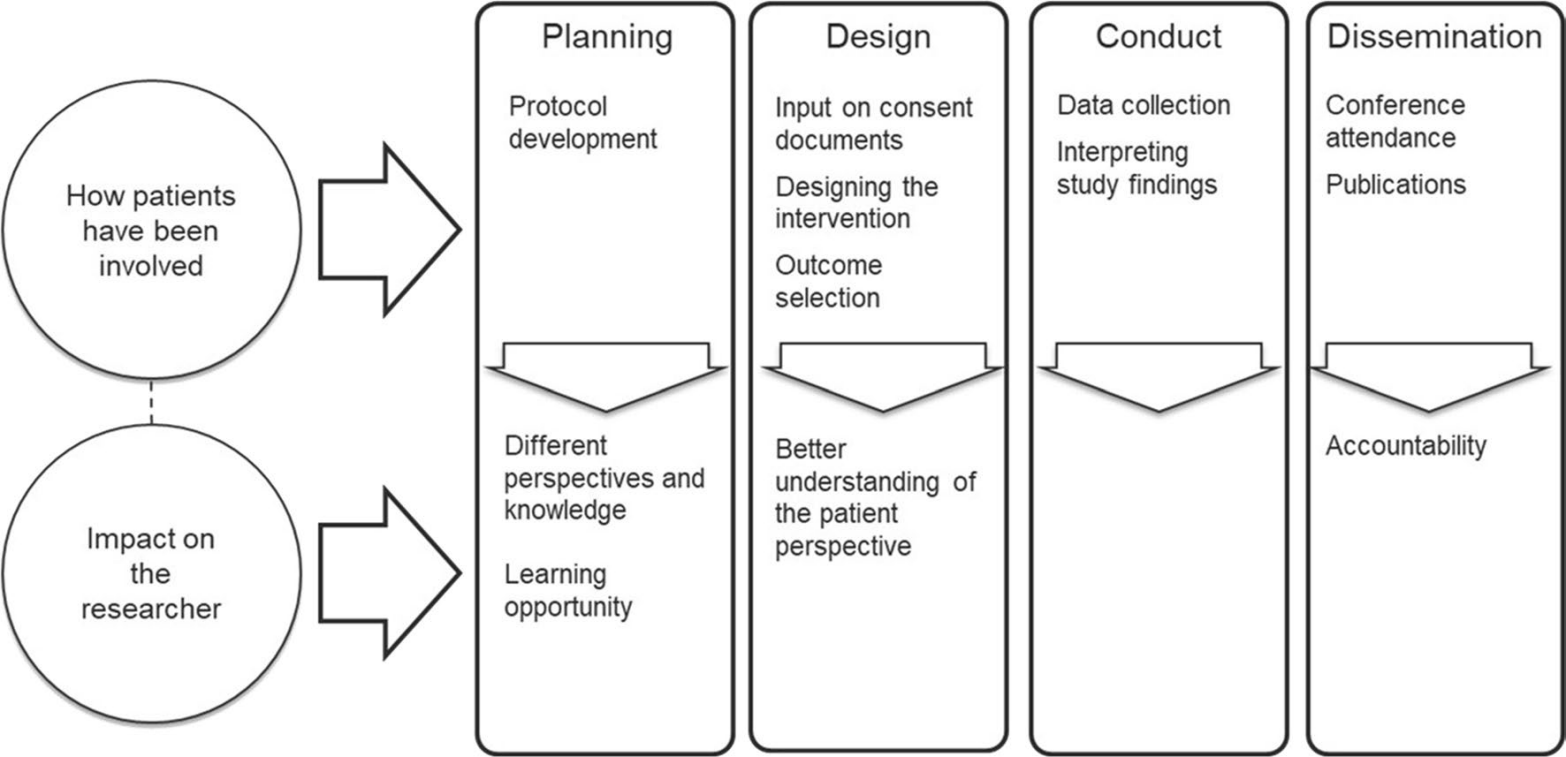
Examples of how patients had been involved and impact on the researcher

Figures 1 and 2 provide an overview of the ways in which interviewees described the impact of patient engagement on their study. Notably, interviewees discussed two major areas of impact: (a) impact on the study about which they were being interviewed, and (b) impact on researchers and research teams which led to changes in practice or approaches in future research.

*3.2.a Impact on the research study: improved relevancy*

Figure 1 provides examples of study phases and how patients had been involved, and examples of the impact that patient engagement was reported to have had on the study. Specific changes or influence varied. Examples included influence on the outcome measured (Quotes 3.2a.1–3.2a.3), better understanding of the burdens patients face and adapting the recruitment process (Quote 3.2a.4), or even through the co-creation of the intervention itself (Quote 3.2a.5–3.2a.6).

When asked how patient engagement had affected the quality of their research, interviewees agreed it had improved the research. Yet a number of interviewees highlighted a lack of clarity in regard to measurements available to assess the benefits of patient engagement to research activities. As one participant put it:

“I think one of the issues that is not resolved for me are what are the—how can we measure that question? How are we to understand the quality—the impact on quality from patient engagement?” *P1, Principal Investigator*

For others, the improvement in quality was captured by impact. Responses from these interviewees included phrases such as “that your intent matches your effect” and keeping research “real and anchored in [the] real world”. Indeed, while distinct impacts such as those above were noted by individual interviewees in relation to their project, the cross-case analysis indicated a common underlying theme; that engagement with patient partners improved the relevancy of the study to participants. As one interviewee put it:

“Certainly, the relevancy of the project itself [improved], I think - if you measure that in a qualitative way […] how the project changed and morphed based on patient input.” *P3, Principal Investigator*

However, higher levels of engagement or co-creation of research could, paradoxically, make it more challenging to demonstrate the specific impact or effect that the patient engagement had on the study. For example, when asked to share how patient engagement had changed their study, one participant challenged the framing of the question, instead arguing that because the study had been co-created it was impossible to parse out the influence of the patient partners from those of the researchers and other team members.

*3.2.b Impact on the researcher and research team*

In addition to the influence that patient engagement had on the specific design or conduct of the study, the researchers among the pool of participants noted how patient engagement had changed the way that they did research (Quote 3.2b.1). Figure 2 provides examples of how patients had been involved and examples of the impact on the researchers. Specifically, researchers noted that by working with patient partners they had an increased awareness of the issues faced by patients and thus had adapted or reoriented their focus (Quote 3.2b.2). Others noted that patient engagement had changed their practice by providing an accountability measure; they were held accountable to follow through on decisions made. Indeed, a noted impact was that the experience with patient engagement had led to a commitment to patient engagement in future research projects (Quote 3.2b.3), as one participant noted:

“Even for me, I've had a few other grants that I've applied for and been successful with since then where we've done exactly that [had patient engagement], and right from the get go, right from writing the grant I've had youth on that grant helping me write that grant, and tear it up, and help create the product that happens with that. As a result, I've been successful, and I think that wouldn't have happened. I don't think I would have built that into my process in the same way without this experience through the IMPACT Awards, certainly.” *P9, Principal Investigator*

Yet it was also understood that this impact of engagement was a process of ongoing learning and may have varied effects in future work (Quote 3.2b.4).

### Challenges

Participants identified challenges to patient engagement. These included: (a) identifying and reaching patient advisors and patient partners, (b) time-related challenges and, (c) maintaining engagement.

*3.3.a Identifying and reaching patient advisors and patient partners*

For several of the interviewees, an initial challenge was identifying who would have relevant expertise to serve as a patient partner or advisor. Common factors that were raised as relevant to the decision included experience with the health condition under study, or experience with the health system in question.

For example, interviewees noted that when the research question centred around a particular health condition, then it may be more straightforward to identify a patient population from whom they could seek patient partners or advisors. However, in areas such as public health or other areas that were not condition-specific, the identification of relevant expertise or characteristics could be more challenging (Quote 3.3a.1). In other instances, there were more practical issues, such as a short period of ill health or contact with the health system, that meant that there was potentially limited engagement with the issue at hand and thus less saliency with the research:

“That's a bit of a challenge…to identify very motivated folk if you're studying a condition that did not impact or affect them long term. Again, we're going back to challenges of studying a population that is not a chronic—suffering from a chronic disease, and for which the impact is temporary and not life altering. That continues to be a bit of a challenge.”

*P3, Principal Investigator*

Others reflected on the input of individuals without experience of the issue being studied, with one participant noting:

“I think a lot of when we were planning this out, we thought the feedback we should be getting from families or people who have experienced [health condition], or are passionate about [health condition]—and I think what I've learned is maybe that doesn't have to be the case, and maybe you just need to go to venues where you have really passionate people who want to help and sort of change the world in their own way. I think we benefit from that.” *P9, Principal Investigator*

A sub-theme related to who the groups of patient partners were deemed to represent and whether those engaged reflected the range of opinion (Quote 3.3a.2). Specifically, interviewees reflected on the diversity and inclusiveness of the patients who were engaged and the degree to which they reflected the patient population served by the study. For example, some interviewees reflected on how financial barriers may mean only those who were financially stable could participate as a patient partner with the resulting input on study design potentially not acknowledging challenges faced by those who are less financially secure. Reflecting on this perceived need for diversity within the patient partners engaged, one interviewee stated:

“I think that having representation of [a] diverse perspective—diverse in terms of background, patient experience, so having mental health is really great, because that ended up being one of our prime—the primary referral, but I think having diverse in terms of age, and perhaps medical condition, as a caregiver, as a family member, as a patient themselves, and having more patients at the table, so that when people aren't able to come because of health reasons, having one patient, and 15 others around the table, there's an imbalance here.” *P4, Research Staff*

As part of this discussion about reach and representation, the issue of compensation was raised. In some instances, this was raised as a challenge; some projects may not have funding available to support financial compensation, or there may be a lack of clarity regarding levels of support or how payments would be managed (Quotes 3.3a.3–3.3a.4). However, it was also raised as a way in which researchers could break down some of the barriers to engagement and reach a more diverse group:

“It's unlikely that […] parents feel that they're going to achieve direct benefit from participation. In that context, we really—I think it's important to add that we have distribution of different voices and perspectives, and I think unless you provide incentives for people to participate in such a thing, and I still feel like the people, that the people who are participating are likely not necessarily representative of the entirety of our cohort. When I've reviewed the literature on how to improve participation in marginalised communities, it is, cash or direct compensation is one of the most effective strategies. To be able to provide that compensation is really important, and we did not include budget for that in our grant, so I think that we are doing [that] now [going] forward.” *P1, Principal Investigator*

*3.3.b Time-related challenges*

A second set of challenges identified were time related. This included awareness of, and accounting for, existing time commitments that patient partners have, understanding that patient engagement may mean that additional time is needed for research, and that engagement with age-bound groups may affect ongoing engagement (Quotes 3.3b.1–3.3b.3).

Discussion about recognising the existing time commitments of patients and family members was commonly raised in the context of how the team had attempted to facilitate patient involvement: participants noted the need for researchers to move out of their own domain, or ‘ivory tower’ (Quote 3.3b.4) and provide supports to facilitate patient engagement (Quote 3.3b.5). For example, one participant noted how parents would use social media groups to interact with each other. The research team, therefore, used social media and these groups to engage parents. This allowed parents to respond in their own time, which may be outside office hours, rather than having to fit around the research teams’ schedule:

“I think the next piece is to be thoughtful that there are many different ways that a patient can contribute to research, and we need to meet—we need to consider how do we meet patients where they are, and where they want to be, and how they want—and really tailor those opportunities to how they want to contribute, because it's all going to be meaningful, but I don't think we should expect all patient leaders to be the same, and to contribute in the same way.” *P9, Principal Investigator*

Indeed, when asked to suggest advice for other researchers, building in extra time for research was a key theme. Not only in terms of allowing longer for individual research to be conducted but acknowledging that the relationships between researchers and patient partners or advisors themselves require an investment of time (Quote 3.3b.6).

*3.3.c Maintaining engagement*

A further time-related challenge was of maintaining engagement in the context of studies where inclusion or relevance was perceived to be time-limited. For example, interviewees whose projects involved children, youth, or young adults reflected on how their patient partners may “age out” or that the study would become less relevant to them as the patient partners grew and where the study was not as immediately relevant to them (Quote 3.3c.1). The challenge of maintaining engagement with patient partners also arose when conditions were transient in nature. For example, a condition would resolve, and the study would again become less relevant to the patient partner.

Maintaining engagement was also a challenge for research-related reasons. Specifically, it was noted that research involved periods of more or less intense activity (Quote 3.3c.2). Maintaining engagement during those lulls in activity was identified as a key challenge for patient engagement.

“[P]art of the challenge of maintaining the engagement with our patient partner so to speak was that there was very little reason to stay engaged with that patient during the one-year duration of the study itself. They participated in steering committee meetings, and they were made aware of the progress of the study, but we had little to ask of them, and they have little to comment upon” *P3, Principal Investigator*

Some researchers did note that they had taken steps to maintain communication, such as maintaining mailing lists, in order to try and keep engagement going during lulls in activity.

## Discussion

In the present study we interviewed ten individuals involved with studies that had engaged patients in some capacity as a requirement of funding. The level of engagement, areas where input was sought, and approaches used were highly diverse, reflecting the perspective—as indicated by one respondent—that patient engagement needs to be in line with the study and thus differs between studies depending on their goals. However, few respondents noted that their work was informed by an underlying theory or framework.

Through our cross-case analysis, we noted the theme of ‘relevancy.’ Based on the context of the examples given we suggest that relevancy be considered as a conceptual underpinning of the impact that patient engagement has on research. While some authors have argued that patient engagement in research can improve the relevance of research through the prioritisation of topics important to patients [17, 18], and others have invoked relevancy to justify the selection of patient-oriented outcomes [19], we suggest that relevancy be construed as a more holistic construct, not merely whether the study addresses a pertinent question or collects meaningful outcomes.

Following Hjørland and Christensen [20] and Hjørland [21], we suggest that relevance is defined in relation to the likelihood of accomplishing the goal at which a task is directed. For example, a trial becomes more relevant if its design and conduct increase the likelihood that the evidence generated is directly applicable to the issue under study. In this context relevancy should be considered a latent construct (one that cannot be directly measured) that is evidenced through other indicators, such as changes to instrumental aspects of the study. We might assume this to be a positive relationship, so more patient input increases study relevancy. For example, patient involvement in designing study materials for a trial may increase their relevancy to patients participating in the trial. This might not only influence initial recruitment but may also affect retention if the materials better convey the goals of the study or the importance of staying in the trial to ensure high-quality data. We suggest, therefore, that relevancy is a central component to understanding how patient engagement works and that it may serve as a useful conceptual underpinning for tools that can promote discussion between patient partners and other stakeholders and inform the research question, design, conduct, and analysis of a study. Of course, in practice operationalising changes to make a study more relevant may be contested. As Hjørland notes:

“What in practice causes problems are theoretical disagreements on what the goals are, on what criteria for good solutions are, and on what methods are available. Goals and problems may be differently conceptualized and connected to different world views and epistemologies.” [21]

Thus, there remains work to build out this conceptual underpinning, and gain deeper insights from patient perspectives, in order to develop tools that can assist in operationalising the concept of relevancy with respect to health research. Here the work to develop pragmatic randomised controlled trials (RCTs) may prove useful. Pragmatic RCTs study effectiveness of an intervention under ‘real-world’ circumstances with the goal of providing information that can directly inform a health care or health policy decision. The interest in pragmatic RCTs led to the development of tools such as the Pragmatic-Explanatory Continuum Indicatory Summary (PRECIS-2) [22] which assist investigators in matching specific design features to the intended study question/clinical decision. We suggest tools such as the PRECIS-2 framework [22] could be useful starting points for developing tools to operationalise the concept of study relevancy.

Secondly, we note the reported longer-term impacts of patient engagement, and specifically the impact noted on researchers and research teams. These were sometimes manifested as awareness of clinical realities that could be accounted for in future research, but other influences related to the process of patient engagement and awareness of the dynamics at play within the study team. This finding is in line with the idea of experiential knowledge insofar as the researcher gains understanding through their experience of working with patients as partners or advisors [23, 24] but also facilitates the development of ongoing patient-researcher relationships. This long term aspect potentially poses challenges to frameworks for evaluating the impact of patient engagement, and which tend to be centred around short-term impacts on the immediate study [7, 25–27], as opposed to capturing longer-term impact that might go beyond the life of any one specific project [28]. We suggest that evaluation of the longer impact of patient engagement, potentially extending over multiple projects and incorporating how relationships with patient partners have evolved, is an area in need of further exploration.

The challenges identified by interviewees; identifying and maintaining engagement with patient partners, building relationships, diversity of patient partners, and challenges of time, resonate with those noted in the literature [24, 29–34]. Indeed, many of the challenges and mitigation strategies within our study are the same as those that have been reported in numerous studies, suggesting that many lessons remain to be learned. Moreover, despite our focus on studies within Ontario, Canada, the findings are consistent with international research, such as the work of Heckert et al., who collected data from PCORI investigators in the US. In their work they identified a number of ‘infrastructural challenges’ including the need for substantial time and effort to support partners and manage engagement; challenges in identifying partners with diverse backgrounds and perspectives; and, challenges in maintaining consistent partner participation [33]. This suggests that while our focus was regional, the issues identified are more general to research in which patients are engaged as partners and would benefit from national or international research.

There were also notable differences between our findings and those of previous studies. While previous studies have noted concerns and challenges about the perceived representativeness of patient partners (in terms of any one person representing a whole population) [5], the focus of concern among our interviewees tended to be the identification of appropriate patient partners or advisors and acknowledging the need to have diversity in those partners. Financial compensation has been noted as both a facilitator to participation, by overcoming financial barriers [30] (as suggested here), but also as a potential challenge in itself (due to difficulties processing payments or due to the impact that payment may have on existing benefits received by individuals) [35]. Indeed, compensation—financial and non-financial—has become a major topic of discussion in the patient engagement literature [36]. This includes both renumeration for expertise and time as well as compensation and resources to address barriers to engagement such as the provision of necessary technology or equipment and childcare or elder care. Comments from interviewees in our study suggest this will be an ongoing topic for discussion with ongoing challenges regarding when compensation should be considered, levels of compensation that may be appropriate, and practical challenges faced when implementing compensation policies [37].

This work is not without its limitations. First, our sample is drawn from research teams who applied for funding that specified the need for patient engagement and where teams had access to methodological support services offered by the SPOR SUPPORT Unit. The likely self-selection of research teams, and infrastructure support available, should be kept in mind given that these supports may not be broadly available. Further, interviews tended to be conducted with the principal investigators. While these individuals are the study figurehead, they may not have as much day-to-day interaction with the patient partners, nor the implementation of the research. As such, had we had more interviewees who were research staff or patient partners, we may have identified different effects, and additional challenges to patient engagement. Furthermore, recruitment of patient partners was mediated by the principal investigator, and we are unable to explore the degree to which investigators shared the study information and the degree to which patient partners themselves declined to participate. A strength of the present manuscript, however, has been the inclusion of patient partners from the outset and who have informed the study design and analysis bringing many years of partnership to bear on their reflections and which has provided essential contextualisation for our findings. A second limitation is that all interviewees were from studies that were clinical trials. Other types of study design may generate novel challenges not noted here. Finally, all interviews were conducted in English, which may have precluded identification of additional issues that may arise with linguistic diversity or that may be faced by individuals or populations whose principal language is not English. However, the consistency of issues identified here with those across the international literature point to the validity of findings.

## Conclusion

Our analysis points to relevancy as a conceptual underpinning to the instrumental impact that patient partners and advisors can have on the immediate study design. However, there remains a need to further build out this concept and how it may be operationalised in practice. Further, the longer-term impacts of patient engagement on researchers and research terms remains under-explored and may reveal additional elements for evaluation, notably the development of ongoing relationships between research teams and patient partners. However, challenges to patient engagement remain, including identifying and maintaining engagement with partners that reflect the diversity of the population of interest. Developing approaches and interventions to address these challenges remains a priority.

## Supplementary Information

**Additional file 1.** Interview guide.

## Data Availability

The datasets generated and/or analysed during the current study are not publicly available due potential identifiability.

## Abbreviations

CIHR: Canadian Institutes of Health Research
OSSU: Ontario SPOR SUPPORT Unit (OSSU)
IMPACT: Innovative, Measurable, Patient-oriented, Appropriate, Collaborative, and Transformative
PCORI: Patient-Centered Outcomes Research Institute
PI: Principal investigator
PRECIS: Pragmatic-Explanatory Continuum Indicatory Summary
SPOR: Strategy for Patient Oriented Research

## Footnotes

1 *The term “public involvement” is most commonly used in the UK to describe the meaningful and active collaboration between patients and the public in research. This will be used synonymously here with the Canadian term “patient engagement”.*
