# Supplementary material for: The impact of patient engagement on trials and trialists in Ontario, Canada: An interview study with IMPACT awardees
Source: Res Involv Engagem. 2022 Sep 7;8:50. doi: 10.1186/s40900-022-00381-7 (PMC9450365; doi:10.1186/s40900-022-00381-7)
Supplement: Supplementary file 1 — Additional file 1. Interview guide. [file 40900_2022_381_MOESM1_ESM.docx]

**SUPPLEMENTARY FILE S1**

## **Topic Guide (Modified from INVOLVE, 2013 for SPOR work)**

How has patient engagement impacted on your SPOR-funded health research?

1. A brief summary of the research you have been doing
   - Did you have a particular theoretical approach or framework to guide the patient engagement activities of the research—and if so, what was it?
2. How were patient partners engaged in the research?
   - How many?
   - At what stages?
   - In what roles?
   - What methods? (Focus group, steering committee, advisory, etc.)
3. Have your patient engagement approaches been successful?
   - If so, to what do you attribute this success?
   - If not, what challenges did you face? Were you able to address them? If yes how, if not, why not?
4. What difference(s) do you think patient partner engagement made to the research?
   - Both positive and negative
5. How do you think the research was changed by patient engagement?
   - Can you say how you know this—e.g. give examples of changes made as a result of patient engagement
6. Do you think patient engagement made a difference to the quality of the research?
   - Can you say a bit more about your understanding of ‘quality’ in research in this context?
7. What difference were you hoping patient engagement would make to the research?
   - Was there anything unexpected or surprising about the impact of patient engagement on the research?
8. Can you say if patient engagement had an impact on the implementation of the research?
9. Did you find that training patient advisors/partners was necessary?
   - Did you offer training?
   - Would you recommend training of patient advisors/partners?
   - Did you find that patients were comfortable sharing their candid opinion?
10. *FOR RESEARCHERS:* How experienced were you in involving patients in your own research prior to this funded research? (In what ways had you involved them previously?)
    - And how did this past experience affect how you approached working with patients for this project? (repeated similar, tried new things)

*FOR PATIENTS:* Have you ever partnered on a research team before?

- And how did this past experience affect how you approached working with researchers for this project? (any expectations or new approaches?)

1. *FOR RESEARCHERS:* Given your experience in your present project, what were the key factors for success that you experienced that you wish you would have known going into this, that might help other researchers seeking to involve patients more in their research
   - Can you state any barriers or enablers to patient engagement that you encountered? Did anything surprise you in involving patients in your research?

*FOR PATIENTS:* Given your experience in your present project, what were the key factors for success that you experienced that you wish you would have known going into this, that might help other researchers seeking to involve patients more in their research

- Can you state any barriers or enablers to patient engagement that you encountered?

1. Any final thoughts or lessons learned?
